# Low incidence of venous thrombosis but high incidence of arterial thrombotic complications among critically ill COVID-19 patients in Singapore

**DOI:** 10.1186/s12959-021-00268-9

**Published:** 2021-03-08

**Authors:** Chuen Wen Tan, Bingwen Eugene Fan, Winnie Z. Y. Teo, Moon Ley Tung, Humaira Shafi, Dheepa Christopher, Shuwei Zheng, Wee Ming Peh, Stephrene Seok Wei Chan, Vanessa Cui Lian Chong, Christian Aledia Gallardo, Cheng Chieh Ray Chang, Li Min Ling, Jing Yuan Tan, Ken Cheah Hooi Lee, Ghee Chee Phua, Benjamin Pei Zhi Cherng, Jenny Guek Hong Low, Vui Kian Ho, Vishnu Prasad, Lester Jung Long Wong, Cheryl Xiu Qi Lim, Yen Lin Chee, Kiat Hoe Ong, Lai Heng Lee, Heng Joo Ng, Eng Soo Yap, on behalf of the Thrombosis Haemostasis Workgroup of Singapore Society of Haematology

**Affiliations:** 1 Department of Haematology, Singapore General Hospital, Singapore, Singapore; 2 Department of Haematology, Tan Tock Seng Hospital, Singapore, Singapore; 3 Department of Laboratory Medicine, Khoo Teck Puat Hospital, Singapore, Singapore; 4 Department of Haematology-oncology, National University Cancer Institute, Singapore, Singapore; 5 Fast Program, Alexandra Hospital, Singapore, Singapore; 6 Department of Infectious Diseases, Changi General Hospital, Singapore, Singapore; 7 Department of General Medicine, Sengkang General Hospital, Singapore, Singapore; 8 Department of Intensive Care Medicine, Sengkang General Hospital, Singapore, Singapore; 9 Department of Infectious Diseases, National Centre for Infectious Disease, Singapore, Singapore; 10 Department of Respiratory and Critical Care Medicine, Singapore General Hospital, Singapore, Singapore; 11 Department of Infectious Diseases, Singapore General Hospital, Singapore, Singapore; 12 Programme in Emerging Infectious Diseases, Duke-NUS, Singapore, Singapore; 13 Division of Medicine, Khoo Teck Puat Hospital, Singapore, Singapore; 14 Department of Medicine, Ng Teng Fong General Hospital, Singapore, Singapore; 15 Department of Laboratory Medicine, National University Hospital, Singapore, Singapore

**Keywords:** COVID-19, Thrombosis, Critical care

## Abstract

**Background:**

Arterial and venous thrombosis are reported to be common in critically ill COVID-19 patients.

**Method and results:**

This is a national multicenter retrospective observational study involving all consecutive adult COVID-19 patients who required intensive care units (ICU) admission between 23 January 2020 and 30 April 2020 in Singapore. One hundred eleven patients were included and the venous and arterial thrombotic rates in ICU were 1.8% (*n* = 2) and 9.9% (*n* = 11), respectively. Major bleeding rate was 14.8% (*n* = 16).

**Conclusions:**

Critically ill COVID-19 patients in Singapore have lower venous thromboembolism but higher arterial thrombosis rates and bleeding manifestations than other reported cohorts.

## Introduction

COVID-19 is associated with hypercoagulability [1] and a high incidence of thrombotic complications in critically ill patients [2]. Initial reports in Western populations suggest thrombotic rates as high as 49% despite thromboprophylaxis [3] while deep vein thrombosis (DVT) rate of 46% have been reported by the Chinese [4]. These disconcerting findings have prompted suggestion for empiric escalation of prophylactic anticoagulation therapy [3] but expert consensus [5, 6] and more recent data have questioned this rationale [7]. Of concern is hypercoagulability overlapping with sepsis-induced coagulopathy and thrombotic microangiopathy, resulting in a dynamic haemostatic environment with higher potential for bleeding complications from interaction with pharmacological thromboprophylaxis. Accurate profiling of thrombotic and bleeding complications in these patients is paramount for optimal case management and outcomes.

This study describes the thrombotic and bleeding manifestations among critically ill COVID-19 patients in Singapore.

## Methods

This multi-center retrospective cohort study involved all eight public general hospitals with intensive care units (ICU) in Singapore-Alexandra Hospital (AH), Changi General Hospital, Khoo Teck Puat Hospital, National University Hospital, Ng Teng Fong General Hospital, Tan Tock Seng Hospital/National Centre for Infectious Disease campus, Sengkang General Hospital and Singapore General Hospital. From 23 January 2020 through 30 April 2020, all adult patients with COVID-19 confirmed by a respiratory SARS-CoV2 RT-PCR test and were admitted to any of the ICUs were identified. The study protocol was approved by the centralized institutional review board covering all participating hospitals (protocol no. CIRB 2020–2528) except AH which only contributed data on the total number of COVID-19 ICU admission and thrombotic events. Anonymized data was provided by each participating site and pooled for analyses. Laboratory results were based on the first results available when the patients were admitted to ICU. The primary outcome was any venous or arterial thrombotic event in the ICU. Other measures included (1) any thrombotic events throughout the period of hospitalisation, (2) major and minor bleeding events during hospitalisation, (3) factors associated with thrombotic and bleeding outcomes and (4) mortality. Venous thromboembolism (VTE) was diagnosed based on clinical suspicion and confirmation by Doppler ultrasound of the extremities or computed tomography. Myocardial infarctions (MI), type I ST elevation and non-ST elevation MI, were diagnosed based on dynamic changes in cardiac enzymes and electrocardiogram while ischaemic strokes were confirmed by MRI scans. Diagnosis of these arterial events were verified by attending specialists (cardiologists and neurologists respectively). Bleeding complications were graded using the modified World Health Organization (WHO) grading system [8]. Each hospital has its own standardised ICU bundles in which patients are started on pharmacological prophylaxis. However, the attending physicians are allowed clinical discretion and those patients deemed not suitable for pharmacological thromboprophyalxis are put on mechanical prophylaxis.

Descriptive statistics were used to analyse continuous and categorical variables. Logistic regression was used to evaluate potential risk factors for the secondary outcomes. All data analyses were performed using SPSS version 23.0 (IBM, USA).

## Results

One hundred eleven COVID-19 patients were admitted to the ICUs during the study period. The overall thrombotic rates in ICU were 11.7% (95% confidence interval (CI):7.0–19.0%) (*n* = 13) with 1.8% (95% CI: 0.5–6.3%) (*n* = 2) venous and 9.9% (95% CI: 5.6–16.9%) (*n* = 11) arterial events. Corresponding rates throughout hospitalisation, censored at 30 April 2020, were 18.0% (95% CI: 12.0–26.2%) (*n* = 20) with 6.3% (95% CI: 3.1–12.5%) (*n* = 7) venous and 11.7% (95% CI: 7.0–19.0%) (*n* = 13) arterial events. After the exclusion of cases from AH (*n* = 3, no thrombotic events), the remaining 108 patients contributed a total of 311.4 patient-weeks for further analysis (Table 1). As of 30 April, 70 patients (64.8%) had been discharged while 9 had died (8%) and 30 (27.7%) were still hospitalized.

**Table 1:** Clinical characteristics and laboratory findings of 108 critically ill COVID-19 patients stratified according to their thrombosis status

Characteristics	All patients (***n*** = 108)	No thrombotic events (***n*** = 88)	Venous thrombosis (***n*** = 7)	Arterial thrombosis (***n*** = 13)
**Demographics**				
Age (years)	62 (19–88)	61.5 (19–88)	62 (54–75)	64 (40–82)
Male	75 (69.4%)	59 (67%)	5 (71.4%)	11 (84.6%)
Body weight > 100 kg	6 (5.6%)	5 (5.7%)	0 (0%)	1 (7.7%)
Ethnicity				
Chinese	67 (62%)	55 (62.5%)	6 (85.7%)	6 (46.2%)
Malay	14 (13%)	12 (13.6%)	1 (14.3%)	1 (7.7%)
Indian	16 (14.8%)	12 (13.6%)	0 (0%)	4 (30.8%)
Thai/Burmese	3 (2.8%)	3 (3.4%)	0 (0%)	0 (0%)
Others	8 (7.4%)	6 (6.8%)	0 (0%)	2 (15.4%)
**Pre-existing medical conditions**				
Major illness				
Hypertension	62 (57.4%)	50 (56.8%)	3 (42.9%)	9 (69.2%)
Ischaemic heart disase	21 (19.4%)	16 (18.2%)	2 (28.6%)	3 (23.1%)
Dyslipidemia	52 (48.1%)	40 (45.5%)	4 (57.1%)	8 (61.5%)
Heart failure	4 (3.7%)	4 (4.5%)	0 (0%)	0 (0%)
Previous stroke	6 (5.6%)	5 (5.7%)	0 (0%)	1 (7.7%)
Diabetes	40 (37%)	35 (39.8%)	1 (14.3%)	4 (30.8%)
Renal impairment	15 (13.9%)	12 (13.6%)	1 (14.3%)	2 (15.4%)
History of venous thromboembolism	4 (3.7%)	4 (4.5%)	0 (0%)	0 (0%)
Anti-coagulant therapy at admission	4 (3.7%)	4 (4.5%)	0 (0%)	0 (0%)
Anti-platelet agent at admission	20 (18.5%)	15 (17%)	2 (28.6%)	3 (23.1%)
**ICU-specific findings**				
APACHE II	11 (0–32)	10.5 (0–32)	11 (6–27)	11 (4–20)
SOFA	3 (0–16)	3 (0–12)	2 (0–16)	4 (1–9)
Mechanical ventilation	84 (77.8%)	66 (75%)	5 (71.4%)	13 (100%)
Dialysis support	28 (25.9%)	21 (23.9%)	2 (28.6%)	5 (38.5%)
Onset of symptoms till ICU admission in days	8 (0–34)	7.5 (0–34)	8 (4–15)	9 (3–34)
Prophylactic anticoagulation	69 (63.9%)	59 (67%)	4 (57.1%)	6 (46.2%)
**Thrombosis-related features**				
Onset of thrombosis from hospital admission in days			13 (7–42)	7 (0–25)
Onset of thrombosis from admission to ICU in days			7 (2–23)	5 (0–13)
**Bleeding complications**				
Major bleeding events	16 (14.8%)	10 (11.4%)	3 (42.9%)	3 (23.1%)
Minor bleeding events	4 (3.7%)	4 (4.5%)	0 (0%)	0 (0%)
**Death**	9 (8.3%)	5 (5.7%)	1 (14.3%)	3 (23.1%)
**Laboratory findings, median (range)**				
PT (s)	12.6 (11.2–13.6)	12.0 (11.0–13.1)	13.4 (13.2–15.3)	12.8 (11.5–15.7)
aPTT (s)	36.9 (32.8–41.9)	36.8 (32.6–41.2)	33.8 (32.6–38.2)	(39.8 (32.9–42.2)
D-dimer (mg/L FEU)	2.3 (1.2–6.1)	1.9 (1.2–5.5)	2.7 (2.4–2.7)	6.1 (1.8–12.8)
Fibrinogen (g/L)	5.9 (4.2–8.2)	6.8 (4.1–8.0)	4.0 (2.1–5.6)	6.2 (4.7–9.2)
White blood count (× 10^9^ /L)	8.5 (6.1–11.7)	8.3 (6.0–11.1)	11.3 (6.5–14.9)	10.5 (7.5–12.9)
Absolute lymphocyte count (×10^9^ /L)	0.9 (0.6–1.5)	0.9 (0.6–1.5)	0.9 (0.6–1.5)	0.9 (0.6–1.7)
Absolute neutrophil count (×10^9^ /L)	7.1 (4.5–10.3)	6.9 (4.5–10.1)	7.9 (5.2–13.8)	8.6 (5.0–11.5)
Plaletet count (×10^9^ /L)	221 (116–350)	219 (161–350)	232 (155–385)	230 (160–338)
CRP (mg/L)	158 (99–249)	174 (97–265)	142 (17–198)	140 (114–163)
Procalcitonin (μg/L)	0.5 (0.2–1.3)	0.5 (0.2–1.3)	0.2 (0.2–2.3)	0.6 (0.2–2.1)
Creatinine (μmol/L)	85 (68–120)	85 (68–120)	78 (62–135)	88 (71–120)

Continuous variables denoted as median (range); categorical variables as number (%)

Two VTE events, comprising a lower limb DVT and a line-related upper limb DVT, were diagnosed in two patients in ICU, giving a VTE rate of 0.6 (95% CI: 0.1–2.3) per 100-person-weeks. For the entire duration of hospitalization, the cumulative VTE rate rose to 2.2 (95% CI: 0.9–4.6) per 100-patient-weeks. Of these, the majority were pulmonary embolism (Table 2). 75% of the patients received therapeutic anticoagulation after the diagnosis of VTE with 2 subsequently stopped due to bleeding complications.

**Table 2:** Description of the thrombotic and bleeding cases

Type of event	Number of cases	Details
Pulmonary embolism (PE) only	4	4 cases of PE diagnosed on CTPA
PE and proximal lower limb deep vein thrombosis (DVT)	1	1 case had both PE and DVT
Proximal lower limb DVT only	1	1 case had both proximal lower limb DVT
Other venous thromboembolism sites	1	1 case had line related upper limb DVT
Myocardial infarction (MI) only	11	11 cases had MI
MI and ischaemic stroke	1	1 case had both MI and ischaemic stroke
Ischaemic stroke	1	1 case had ischaemic stroke
Bleeding (Major)	16	1 Intracranial haemorrhage – while on IV prophylactic heparin for CVA
		2 cases of intracranial haemorrhage – while on ECMO with IV unfractionated Heparin
		1 PR bleeding from haemorrhoids/anal fissures – while on therapeutic LMWH for PE
		1 PR bleeding from haemorrhoids – while on prophylactic LMWH
		1 PR bleed from haemorrhoids – not on anticoagulation
		1 Severe gastritis with melena – while on S/C therapeutic LMWH for DVT
		1 Recurrent Forrest 2C ulcer bleed – while on S/C therapeutic LMWH for DVT
		1 Stress gastropathy with nasogastric tube erosion and worsening anaemia – not on anticoagulation
		1 Coffee ground NG aspirate related to celecoxib use
		1 Coffee ground NG aspirate – not on any anticoagulation
		1 Gastrointestinal bleed – not on any anticoagulation
		1 Haemorrhagic encephalitis and PR bleed – while on prophylactic LMWH
		1 Haemoptysis – while on prophylactic LMWH
		1 Bloody trachea aspirates – while on IV unfractionated Heparin for STEMI
		1 Bleeding from lines – while on IV unfractionated Heparin for IVC thrombosis
Bleeding (Minor)	4	2 cases of blood stained sputum – while on prophylactic LMWH
		1 Mild haematuria after urinary catheter insertion – while on apixaban prophylaxis
		1 Mild bleeding from central venous catheter – while on prophylactic LMWH

Acute pulmonary embolism was diagnosed with CT-pulmonary angiography, deep vein thrombosis/upper extremity vein thrombosis was diagnosed with ultrasonography, strokes were diagnosed with CT

*CT* computed topography, *ECMO* Extracorporeal membrane oxygenation, *IV* intravenous, *LMWH* Low molecular weight heparin, *NG Aspirate* Nasogastric aspirate, *S/C* subcutaneous, *STEMI* ST Elevation MI

The arterial thrombosis rate during ICU stay was 3.5 (95% CI: 1.8–6.3) per 100-patient-weeks. This increased marginally during the entire hospitalization to 4.2 (95% CI: 2.2–7.1) per 100-patient-weeks. These events were mainly MI of which one was fatal (Table 2).

The overall thrombotic complication rate in these 108 patients was 6.4 (95% CI: 3.9–9.9) per 100-patient-weeks. 46.2% patients were receiving pharmacological thromboprophylaxis at the time of the events.

The major bleeding (WHO grade 3–4) rate was 5.1 (95% CI: 2.9–8.3) per 100-patient-week. (Table 2) with an overall bleeding rate was 6.4 (95% CI: 3.9–9.9) per 100-patient-days. One bleeding event, from an intracranial hemorrhage, was fatal.

Whilst no clinical factor was significantly associated with the occurrence of thrombotic events, the need of haemodialysis support in ICU and higher fibrinogen level were respectively associated with higher and lower risk for major bleeding events (Table 3a). Mortality was associated with thrombosis but not bleeding (Table 3b).

**Table 3:** (a) Odds ratio of clinical and laboratory factors for thrombotic (arterial and venous) and major bleeding events. (b) Association of thrombotic and major bleeding events to mortality

a) Factors	Thrombotic Events				Major Bleeding Events			
	Odds Ratio	95% Confidence Interval		***P*** value	Odds Ratio	95% Confidence Interval		***P*** value
		Lower Bound	Upper Bound			Lower Bound	Upper Bound	
**Baseline demographics**								
Age	1.034	.990	1.080	.13	1.026	.980	1.075	.27
Gender, male	1.966	.603	6.414	.26	1.381	.410	4.650	.60
Ethnicity, Chinese	.900	.333	2.430	.84	.754	.257	2.207	.61
Diabetes	.505	.168	1.514	.22	.740	.237	2.310	.60
Hypertension	1.140	.424	3.065	.80	.704	.243	2.040	.52
Hyperlipidemia	1.800	.670	4.835	.24	1.091	.377	3.155	.87
Renal impairment	1.118	.284	4.399	.87	2.455	.672	8.962	.17
Pre-existing cardiovascular disease^a^	1.667	.562	4.945	.36	.780	.203	2.999	.72
**ICU-specific features**								
APAHCE score	.979	.904	1.061	.61	.997	.918	1.082	.94
SOFA score	1.043	.890	1.224	.60	1.161	.993	1.358	.06
Mechanical ventilation	3.000	.644	13.973	.16	5.000	.626	39.963	.13
Dialysis support	1.718	.606	4.867	.31	4.940	1.629	14.978	.005
Use of thromboprophylaxis	.492	.184	1.313	.16	.376	.128	1.108	.08
**Laboratory findings**								
PT	1.133	.965	1.329	.13	1.060	.906	1.241	.47
aPTT	.999	.967	1.032	.97	1.003	.967	1.041	.88
D-dimer	1.030	.929	1.143	.57	.783	.454	1.352	.38
Fibrinogen	.956	.751	1.217	.78	.658	.453	.957	.03
White blood count	1.037	.962	1.117	.35	.994	.906	1.089	.89
Absolute lymphocyte count	1.246	.698	2.226	.46	.573	.217	1.511	.26
Absolute neutrophils count	1.030	.945	1.123	.50	1.001	.905	1.107	.99
Platelet count	.999	.996	1.002	.60	.998	.995	1.002	.35
C-reactive protein	.995	.989	1.001	.09	1.001	.995	1.006	.75
Procalcitonin	.996	.969	1.023	.76	.937	.776	1.132	.50
Creatinine	1.000	.996	1.004	.86	.999	.995	1.004	.78
**b) Factors**	**Mortality**							
Thrombotic events	4.150	1.004	17.161	.05				
Arterial events	4.450	.961	20.599	.06				
Venous events	1.937	.207	18.141	.56				
Major bleeding events	3.308	.735	14.878	.12				

^a^Included ischemic heart disease, congestion heart failure and stroke

## Discussion

Although only two-thirds of our critically ill COVID-19 patients received thromboprophylaxis, the incidence rate of VTE was only 1.8%. This rate is far lower than similar published studies that included only objectively confirmed symptomatic VTE events [3, 9]. Several reasons could account for the lower VTE rates in our report. Previous studies have shown patients of Asian-descent have lower risk for VTE compared to Western cohorts [10]. Our patients were also younger with fewer comorbidities and they tended to present to the hospital earlier in their course of illness [3, 4, 9], which might have led to earlier interventions as reflected in the low median APACHE and SOFA scores on transfer to ICU.

Of interest, the occurrence of further VTE events after ICU stay suggest the persistence of hypercoagulability [11]. Thromboprophylaxis measures hence should be continued for these patients throughout hospitalisation. However, a more intensified anticoagulation strategy for our patients was negated by the 14.8% major bleeding rate observed, which was considerably higher than other cohorts [7, 12] despite having lesser proportion of our patients on pharmacological thromboprophylaxis. The baseline characteristics of our patients were not notably different from other cohorts (references as above) apart from the ethnic factor in which our patients were predominantly Asian. The potential ethnic difference in bleeding events has also been observed in other settings including reports of higher bleeding rates among Asian patients taking warfarin for atrial fibrillation, compared with non-Asians counterparts [13].

In contrast to VTE, our arterial event rates are high with mainly MIs occurring almost exclusively in the ICU, when the patients were the sickest. Unlike VTE, comparative arterial thrombotic rates in other COVID-19 cohorts are lower at 4% [3, 7] with mainly strokes rather than MIs. Myocardial injury in up to 30% of COVID-19 patients have been reported by some Chinese investigators but this was based on elevation of cardiac troponin levels [14] without verification of MI. Apart from ethnic differences, the baseline cardiovascular risk factors of our patients did not differ notably from existing literature [7] and thus the precise reason behind the higher rates seen in our population is not apparent currently.

This study is limited by its retrospective nature as with most other studies conducted under the present pandemic environment. There was no established imaging protocol for suspected VTE consistent across the hospitals. Similarly, clinical and laboratory data was not uniformly collected and trivial bleeds might have been missed. The small number of thrombotic and bleeding events also limited our statistical analysis of inference.

## Conclusion

Our data is adequately robust to highlight the differences in thrombosis presentations and higher bleeding manifestations compared to other published cohorts. Our findings thus argue against the need for intensification of pharmacological thromboprophylaxis in similar Asian-predominant populations. Use of global coagulation assays [15] in critically ill COVID-19 patients to guide thromboprophylaxis warrant future consideration and exploration. Extended thromboprophylaxis during hospitalisation should also be considered. The role of antiplatelets and low dose direct anti-Xa inhibitors as cardio-protectants should be among future investigations.

## Data Availability

For original data, please contact eng_soo_yap@nuh.edu.sg.
